# The mammalian target of rapamycin contributes to synovial fibroblast pathogenicity in rheumatoid arthritis

**DOI:** 10.3389/fmed.2023.1029021

**Published:** 2023-02-03

**Authors:** Brianne E. Barker, Megan M. Hanlon, Viviana Marzaioli, Conor M. Smith, Clare C. Cunningham, Jean M. Fletcher, Douglas J. Veale, Ursula Fearon, Mary Canavan

**Affiliations:** ^1^Molecular Rheumatology, School of Medicine, Trinity College Dublin, Trinity Biomedical Sciences Institute, Dublin, Ireland; ^2^Translational Immunopathology, School of Biochemistry & Immunology and School of Medicine, Trinity College Dublin, Trinity Biomedical Sciences Institute, Dublin, Ireland; ^3^EULAR Centre of Excellence, Centre for Arthritis and Rheumatic Diseases, St. Vincent’s University Hospital, Dublin, Ireland; ^4^School of Biochemistry and Immunology, Trinity College Dublin, Trinity Biomedical Sciences Institute, Dublin, Ireland; ^5^School of Medicine, Trinity College Dublin, Trinity Biomedical Sciences Institute, Dublin, Ireland

**Keywords:** rheumatoid arthritis, synovial fibroblast, mTOR, hippo-YAP, yes associated protein

## Abstract

**Objectives:**

The mammalian target of Rapamycin (mTOR) is a metabolic master regulator of both innate and adaptive immunity; however, its exact role in stromal cell biology is unknown. In this study we explored the role of the mTOR pathway on Rheumatoid Arthritis synovial fibroblast (RASF) metabolism and activation and determined if crosstalk with the Hippo-YAP pathway mediates their effects.

**Methods:**

Primary RA synovial fibroblasts (RASF) were cultured with TNFα alone or in combination with the mTOR inhibitor Rapamycin or YAP inhibitor Verteporfin. Chemokine production, matrix metalloproteinase (MMP) production, and adhesion marker expression were quantified by real-time PCR, ELISA, and/or Flow Cytometry. Invasion assays were performed using Transwell invasion chambers, while wound repair assays were used to assess RASF migration. Cellular bioenergetics was assessed using the Seahorse XFe96 Analyzer. Key metabolic genes (GLUT-1, HK2, G6PD) were measured using real-time PCR. Reanalysis of RNA-Seq analysis was performed on RA (*n* = 151) and healthy control (HC) (*n* = 28) synovial tissue biopsies to detect differential gene and pathway expression. The expression of YAP was measured by Western Blot.

**Results:**

Transcriptomic analysis of healthy donor and RA synovial tissue revealed dysregulated expression of several key components of the mTOR pathway in RA. Moreover, the expression of phospho-ribosomal protein S6 (pS6), the major downstream target of mTOR is specifically increased in RA synovial fibroblasts compared to healthy tissue. In the presence of TNFα, RASF display heightened phosphorylation of S6 and are responsive to mTOR inhibition *via* Rapamycin. Rapamycin effectively alters RASF cellular bioenergetics by inhibiting glycolysis and the expression of rate limiting glycolytic enzymes. Furthermore, we demonstrate a key role for mTOR signaling in uniquely mediating RASF migratory and invasive mechanisms, which are significantly abrogated in the presence of Rapamycin. Finally, we report a significant upregulation in several genes involved in the Hippo-YAP pathway in RA synovial tissue, which are predicted to converge with the mTOR pathway. We demonstrate crosstalk between the mTOR and YAP pathways in mediating RASF invasive mechanism whereby Rapamycin significantly abrogates YAP expression and YAP inhibition significantly inhibits RASF invasiveness.

**Conclusion:**

mTOR drives pathogenic mechanisms in RASF an effect which is in part mediated *via* crosstalk with the Hippo-YAP pathway.

## Introduction

Rheumatoid arthritis (RA) is a chronic autoimmune disease which primarily affects the joints, mainly of the hands and feet. It is characterized by synovial proliferation, dysregulated angiogenesis and leukocyte infiltration ultimately leading to joint destruction and functional disability (1). One of the primary pathological cell types within the RA joint are the synovial fibroblasts (RASF). RASF are resistant to apoptosis with an increased capacity to proliferate. Moreover, they are also potent producers of proinflammatory cytokines, chemokines, and matrix degradation enzymes, which ultimately results in a highly invasive phenotype that contributes to bone erosion (2, 3). Current treatment approaches target innate and adaptive immune cells, in addition to the cytokines secreted by them (4). However, there is now strong evidence to suggest that a synovial phenotype dominated by RASF may also exist which is refractory to current conventional treatments (5, 6). This underpins the need to understand the molecular signatures and pathways which govern the RASF phenotype to improve treatment outcomes for difficult to treat or refractory RA.

The synovial microvasculature in RA is highly dysregulated with the formation of immature blood vessels, ineffective at supplying a robust oxygen supply to the joint (7). In addition to this, the increased metabolic demands of highly proliferative and inflammatory cells outpace the vascular supply of oxygen thus resulting in a profoundly hypoxic microenvironment (8). We and others demonstrated that hypoxia itself drives mitochondrial dysfunction in RASF, thereby altering cellular bioenergetics and promoting a shift toward glycolysis in the joint (8–11) while the synovial microenvironment itself can alter metabolic dynamics in immune cells (12). mTOR, also known as the mammalian target of rapamycin, is an evolutionarily conserved pathway known for its non-redundant role in cellular metabolism (13). Accumulating evidence now exists confirming that the metabolically demanding processes involved in innate and adaptive immune cell activation are controlled by master regulators of metabolism such as mTOR (14, 15). However, to date, the contribution of mTOR signaling to fibroblast activation and function is almost entirely unknown, with only a handful of studies to date exploring this pathway in RASF. Indeed, globally in the RA synovium, the contribution of mTOR to synovial joint inflammation is also unknown. Studies to date have demonstrated that TNFα induces mTOR activation, potentiating the RASF inflammatory phenotype while mTOR inhibition limits synovial fibroblast invasion and prevents actin reorganization in both DA rat and RA human synovial fibroblasts (16, 17). Moreover, inhibition of mTOR *via* curcumin results in reduced inflammation, synovial hyperplasia, and a reduction in proinflammatory cytokine secretion in CIA rats (18).

The Hippo-YAP pathway is an evolutionarily conserved signaling pathway that regulates cell survival, proliferation, differentiation, tissue development and homeostasis, and organ size control (19). Yes-associated-protein (YAP) along with the PDZ-binding motif transcriptional coactivator TAZ are key downstream effectors within the Hippo-YAP pathway and are important regulators for actin cytoskeletal polymerization and rearrangement and cell motility (20, 21). To date, a large body of evidence exists on the role of the Hippo-YAP pathway in multiple forms of human cancer (22), however, the role of this pathway in autoimmune disease, in particular RA, is yet to be extensively studied. In parallel, limited data also exists on the role of Hippo-YAP on RASF activation and pathogenicity. Recent literature has reported increased expression of YAP in the RA synovium (23) while enriched epigenetic modifications within components of the Hippo pathway have also been reported in RASF compared to OA (24). Furthermore, inhibition of YAP using its specific inhibitor, verteporfin, reduced synovial fibroblast invasion, arthritis severity, and MMP-13 expression in mice (23, 24). Moreover, evidence also exists demonstrating crosstalk between both the Hippo-YAP and mTOR pathways as demonstrated by increased expression of YAP *via* mTORC1 signaling in mouse and human epithelial cell tumors (25). While the YAP pathway itself is also capable of promoting mTOR signaling *via* increased amino acid uptake (26, 27). At present, it is unknown what role mTOR plays in RASF metabolism and pathogenicity and if crosstalk exists between the Hippo-YAP pathway in mediating these effects. Therefore, in the present study, we explored the effect of mTOR inhibition on the metabolism and function of human primary RASF and determined if the Hippo-YAP pathway contributed to these effects.

## Materials and methods

### Patient recruitment and arthroscopy

Patients with active RA were recruited from outpatient clinics at the Department of Rheumatology, St. Vincent’s University Hospital. Arthroscopy of the inflamed knee was performed under local anesthetic, using a 2.7 mm needle arthroscope (Richard Wolf, IL, USA), and macroscopic synovitis was scored. Macroscopic synovitis was assessed under direct visualization at video arthroscopy by the clinician performing the procedure. Macroscopic synovitis was scored using a well-established, validated visual analog scale 1–100 mm as described previously (28). Biopsies were utilized for isolation of primary RA synovial fibroblasts (RASF). Patient demographics, including gender, diagnosis, treatments, serology and DAS28 are described in Supplementary Table 1.

### Isolation of primary fibroblasts

RA synovial biopsies were digested with 1 mg/ml collagenase type II (Worthington Biochemical, Freehold, NJ, USA) in RPMI-1640 (Gibco-BRL, Paisley, UK) for 4 h at 37°C in humidified air with 5% CO_2_. Dissociated cells were grown to confluence in RPMI-1640, 10% FCS (Gibco-BRL), 10 ml of 1 mmol/l HEPES (Gibco-BRL), penicillin (100 units/ml; Bioscience), streptomycin (100 units/ml; Bioscience) and fungizone (0.25 μg/ml; Bioscience) before passaging. Cells were used between passages 3–8.

### Chemokine measurements

To assess the effect of TNFα in the presence or absence of Rapamycin on pro-inflammatory mediators, RASF were seeded in 48-well plates at a density of 3 × 10^4^ and allowed to attach overnight. Cells were incubated in serum-free RPMI-1640 for 24 h and subsequently pre-treated for 2 h with Rapamycin (100 nM) or Verteporfin (50 nM) where indicated before being stimulated with TNFα (1 ng/ml). Supernatants were harvested and levels of IL-8, MCP-1, and RANTES were measured by specific ELISA (MCP-1: eBiosciences, USA; IL-8, RANTES: R&D systems, UK) according to manufacturer’s conditions.

### MMP detection *via* multiplex ELISA

RASF were seeded in 48-well plates at a density of 3 × 10^4^ and allowed to attach overnight. Cells were incubated in serum-free RPMI-1640 for 24 h and subsequently pre-treated for 2 h with Rapamycin (100 nM) or Verteporfin (50 nM) where indicated before being stimulated with TNFα (1 ng/ml). Supernatants were harvested and used to measure MMP-1 and MMP-3 by multiplex ELISA (Meso Scale Discovery) as per the manufacturer’s instructions. Briefly, 25 μl of supernatants was added to precoated multiplex wells for 2 h at room temperature (RT) under constant shaking. Simultaneously, an eight-point standard curve was added to the plate with a lower limit of detection of 11 pg/ml (MMP-1) and 2.1 pg/ml (MMP-3). The plate was subsequently washed three times using PBS/Tween, after which 25 μl of Detection antibody was added for 2 h at RT under constant shaking. Finally, after washing three times, 150 μl of Read buffer was added, and the plate was analyzed using a Meso Sector S600 Imager.

### Transwell invasion assay

Biocoat Matrigel™ Invasion Chambers (Becton Dickinson, UK) were used to assess RASF invasion. Cells were seeded at a density 3.0 × 10^4^ RASF cells per well in the migration chamber on 8 μm membranes pre-coated with Matrigel. Cells were pre-treated with Rapamycin (100 nM) or Verteporfin (50 nM) for 2 h and subsequently stimulated with TNFα (1 ng/ml) for 48 h. Non-migrating cells were removed from the upper surface by gentle scrubbing. Migrating cells attached to the lower membrane were fixed with 4% paraformaldehyde and stained with 0.1% crystal violet. Cells from five random high-power fields for each well were counted to assess the average number of invading cells.

### Migration assay

RASF (2 × 10^4^ cells/well) were seeded in 48-well plates for 24 h and serum starved as previously described above. A single scratch wound was induced through the middle of each well with a sterile pipette tip and cells were subsequently treated with Rapamycin (100 nM) for 2 h followed by stimulation with TNFα (1 ng/ml) for 24 h. RASF migration across the wound margins was assessed and photographed using a phase-contrast microscope. Semi-quantitative analysis of cell repopulation of the wound was assessed. Briefly, cells were fixed with 4% paraformaldehyde, stained with 0.1% crystal violet and the number of migrating cells across the time zero margin was assessed.

### Protein isolation and western blotting analysis

RASF were seeded into 6-well plates and allowed to grow to confluence. Cells were then incubated in serum-free RPMI-1640 for 24 h and subsequently pre-treated, where indicated, with Rapamycin (100 nM) and subsequently stimulated with TNFα (1 ng/ml). Cells were lysed and protein extracted from RASF samples using 100 μL of ice-cold RIPA (Radio-Immunoprecipitation Assay) buffer (Sigma) containing 10 μg/mL phosphatase inhibitor cocktail 3 (Sigma) and 10 μg/ml protease inhibitor cocktail (Sigma). A BCA assay (Pierce Chemical Co., ThermoScientific, Rockford, IL, USA) was used to quantify protein concentration. Protein (5 μg) was resolved by SDS-Acrylamide (4% stacking, 10% resolving) gels were then transferred onto nitrocellulose blotting membrane (Amersham Protran 0.2 μm NC, Cytiva) prior to blocking for 1 h with wash buffer [Tris-buffered saline (TBS) with 0.1% Tween-20] containing 5% bovine serum albumin (BSA) with gentle agitation at RT. Membranes were incubated with rabbit monoclonal anti-YAP (Cell Signaling Technology, 4912S) diluted at 1:1000 in 5% BSA in TBS-Tween-20 at 4°C overnight with gentle agitation. Following three 5-min washes, membranes were incubated with horseradish peroxidase-conjugated anti-rabbit secondary antibody (Cell Signaling Technology, 7074S) diluted at a 1:5000 dilution in 1% BSA in TBS-Tween-20 for 1 h at RT with gentle agitation. β-actin was used as a loading control and identified using monoclonal Anti-β-Actin-Peroxidase antibody (Sigma) diluted at 1:60,000 in 3% BSA in TBS-Tween-20 incubated at RT for 1 h with gentle agitation. The signal was detected using Immobilon^®^ Western Chemiluminescent HRP Substrate (Millipore). Band densities were imaged using the ChemiDoc MP Imaging System (Bio-Rad, USA).

### mRNA extraction and cDNA synthesis

To assess the effects of TNFα stimulation in the presence or absence of Rapamycin on specific metabolic and inflammatory genes, RASF were seeded into 6-well plates and allowed to grow to confluence. Cells were incubated in serum-free RPMI-1640 for 24 h and subsequently pre-treated, where indicated with Rapamycin (100 nM) and subsequently stimulated with TNFα (1 ng/ml). Total RNA was isolated using the miRNeasy Mini Kit (Qiagen, Germany) according to the manufacturer’s protocol. The integrity of RNA samples was assessed using a bioanalyzer (Agilent, CA, USA). Samples with a 260:280 nm ratio of 1.8 and above and an RNA integrity number between 7 and 10 were used in subsequent experiments. Isolated RNA was stored at -80°C. Total RNA (100 ng) was reverse transcribed to cDNA using a high-capacity cDNA reverse transcription kit (Applied Biosystems, Cheshire, UK) and stored at -20°C until further use.

### RT-PCR analysis

Gene expression data were quantified by RT-PCR using the Quant Studio 5 Thermal Cycler (Applied Biosystems, Lewes, UK). Reaction mixtures contained 1 μl of cDNA, SYBR green I PCR mastermix (Applied Biosystems) and target mRNA specific primer pairs as follows:

**Table:** 

Gene name	Forward primer	Reverse primer
RPLPO	5′ GCGTCCTCGTGGAA GTGACATCG 3′	5′ TCAGGGATTGCC ACGCAGGG 3′
HPRT1	5′ ATGGACAGGACTG AACGTCTTG 3′	5′ GGCTACAATGTGA TGGCCTC 3′
HK2	5′ TTCTTGTCTCAGATT GAGAGTGAC 3′	5′ TTGCAGGATGGCTC GGACTTG 3′
G6PD	5′ CGTCACCAAGAACA TTCACGAG 3′	5′ ATGCGGTTCCAGC CTATCTG 3′
GLUT-1	5′ GCCGGCGGAATTC AATGCTG 3′	5′ AGCATCTCAAAGGAC TTGCCCA 3′
VCAM-1	5′ GTAAAAGAATTGCAA GTCTACATATCAC 3′	5′ GATGGATTCACAGAAAT AACTGTATTC 3′
ICAM-1	5′ AACCAGAGCCAGG AGACACTC 3′	5′ GCGCCGGAAAGCT GTAGATG 3′
MMP-1	5′ GCTAACAAATACTGG AGGTATGATG 3′	5′ ATTTTGGGATAACCT GGATCCATAG 3′
MMP-3	5′ CGGTTCCGCCTG TCTCAAG 3′	5′ CGCCAAAAGTGC CTGTCTT 3′

Samples lacking multiscribe reverse transcriptase formed negative controls to ensure target-specific quantification. Data were analyzed using the comparative threshold cycle (Ct) method with normalization to the expression of RPLPO and HPRT1 as endogenous controls.

### Oxygen consumption rate and extracellular acidification rate-seahorse technology

Oxygen consumption rate (OCR) and extracellular acidification rate (ECAR), reflecting oxidative phosphorylation and glycolysis, respectively, were measured before and after treatment with oligomycin (2 μg/ml, Seahorse Biosciences, UK), trifluorocarbonylcyanide phenylhydrazone (FCCP) (5 μM, Seahorse Biosciences) and antimycin A (2 μM, Seahorse Biosciences), and rotenone (2 μM, Seahorse Biosciences) using the Seahorse XF96-analyzer (Seahorse Biosciences). RASF were seeded at 15,000 cells per well in 96-well XF-microplates (Seahorse Biosciences) and allowed to adhere overnight. Following this, cells were pre-treated with Rapamycin (100 nM) for 2 h before being stimulated with TNFα for 24 h. After 24 h cells were rinsed with assay medium [unbuffered Dulbecco’s Modified Eagle’s medium (DMEM) supplemented with 10 mM glucose, pH7.4] before incubation with assay medium for 30 min at 37°C in a non-CO_2_ incubator. Four baseline OCR and ECAR measurements were obtained over 28 min before injection of specific metabolic inhibitors. Moreover, to challenge the metabolic capacity of the RASF, three OCR and ECAR measurements were obtained over 15 min following injection with oligomycin, FCCP, and antimycin A and rotenone. The maximal respiratory capacity and maximal glycolytic capacity were calculated by averaging the three OCR or ECAR measurements following FCCP and oligomycin injection, respectively. The spare respiratory capacity was calculated by subtracting the baseline OCR from the maximal respiratory capacity OCR, and ATP synthesis was calculated by subtracting the post-oligomycin OCR from the baseline OCR. Finally, the extent of proton leakage was calculated by subtracting non-mitochondrial respiration from the minimum rate measurement after oligomycin injection while the coupling efficiency was determined by dividing the ATP synthesis rate by baseline OCR × 100.

### Flow cytometry

RASF were seeded into 6-well plates at a concentration of 1 × 10^5^ cells per well and left to adhere overnight. Following this, cells were incubated in serum-free RPMI-1640 for 24 h and subsequently pre-treated for 2 h with Rapamycin (100 nM) where indicated before being stimulated with TNFα (1 ng/ml). Cells were enzymatically removed from 6-well plates using Accutase and stained as follows. Live Dead Near IR (Biolegend) was used to eliminate dead cells. To eliminate non-specific binding of mouse monoclonal antibodies to the Fc-gamma receptors (FcγR), samples were blocked with a human FcγR-binding inhibitor (TruStain FcX Receptor blocking solution; Biolegend) prior to antibody staining. Cells were then stained as follows: Phospho-S6 PE (Clone 5316S, Cell Signaling Technology); ICAM-1 BV605 (Clone: HA58, BD Biosciences); mTOR AF647 (Clone: O21-404, BD Biosciences); CXCR3 PE (Clone: G025H7, Biolegend); CXCR4 PECF594 (Clone: 12G5, Biolegend); CXCR5 BV786 (Clone: J252D4, Biolegend). In order to adjust for spectral overlap between detectors, compensation was applied using single stained compensation beads (BD). Samples were acquired using the LSR Fortessa (BD) and analyzed using FlowJo software (Treestar Inc.). Cells were gated based on forward and side scatter, and dead cells and doublets were removed. Fluorescence Minus One (FMO) controls were used to determine gating boundaries.

### Bulk RNA sequencing analysis

Transcriptomic analysis was performed on our previously published synovial tissue RNASeq dataset (29). Briefly, quality of RNA was evaluated using an Agilent bioanalyzer followed by RNAseq by Q2 Solutions (Morrisville, NC, USA). Sequencing libraries were prepared on Truseq stranded total RNA using the Illumina Ribo-Zero protocol. Sequencing of pooled libraries was performed on an Illumina HiSeq 2000 and raw read quality was evaluated using FastQC. Raw reads were trimmed based on sequence quality and adaptors leading to an average number of clusters per sample of 8.9 × 107. Reads were then aligned to the human reference genome b37.3 using STAR v2.4 (30). Quantification of aligned reads was performed using RSEM v1.2.14 with the University of California Santa Cruz (UCSC) transcriptome model that included lincRNAs from Ensembl v75. Aligned data were subjected to evaluation of quality utilizing several metrics including mapping rate, coverage, and deviation from PCA. Counts were converted to log2 counts per million, quantile normalized, and precision weighted.

Human donors [healthy, arthralgia, Undifferentiated Arthritis (UA), early RA and established RA] used in this bulk RNAseq analysis have been described previously (29). Briefly, synovial biopsies were obtained from 28 healthy donors (14 male, 14 female) with no evidence of arthritis upon examination or history of arthritis. Arthralgia patients within this cohort (*n* = 10) were defined as individuals with symptoms of aches and pains, without clinical signs of synovitis or significantly raised C-reactive protein but positive for rheumatoid factor (RF) and anti-citrullinated protein antibody (ACPA)+. UA patients (*n* = 6) were defined as individuals presenting with clinical signs of synovitis, but who failed to meet the 2010 American College of Rheumatology criteria for RA. Early RA (*n* = 57) was defined as within 12 months of diagnosis without prior treatment. The mean disease duration in this cohort was 5 months. Established RA patients (*n* = 95) had an average disease duration of 68 months, and all patients were diagnosed > 1 year before the sample collection. All established RA patients had received disease-modifying antirheumatic drugs or anti–TNF-α treatments.

### Statistical analysis

SPSS15 system (SPSS Inc., Chicago, IL, USA) for Windows and Prism 8 software were used for statistical analysis. Wilcoxon Signed Rank test, paired Friedman with Dunn’s Multiple comparisons, or unpaired Mann–Whitney was used for analysis of non-parametric data. *p*-values of less than 0.05 (*p < 0.05) were determined as statistically significant.

### Study approval

Ethical approval to conduct this study was granted by St. Vincent’s Healthcare Group Medical Research and Ethics Committee and all patients gave fully informed written consent prior to inclusion. All experiments were performed in accordance with these guidelines and regulations.

## Results

### Dysregulated expression of the mTOR pathway in RA synovial tissue

**FIGURE 1:**
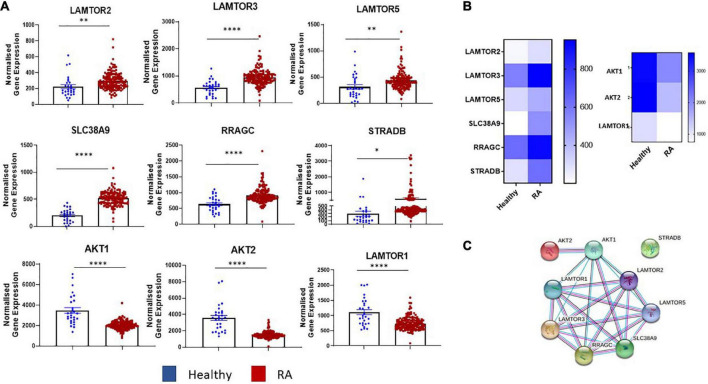
Dysregulated expression of the mTOR pathway in RA synovial tissue. **(A)** Bar graphs demonstrating normalized gene expression of key components in the mTOR pathway (LAMTOR1, LAMTOR2, LAMTOR3, LAMTOR5, SLC38A9, RRAGC, STRADB, AKT1, and AKT2) *via* transcriptomic analysis of RNA-Seq (GEO database GSE89408) on synovial tissue biopsies from healthy donors (*n* = 28) RA patients (*n* = 151). **(B)** Clustered heatmap representing DEG involved in metabolism (mTOR pathway) from RASF compared to healthy controls. **(C)** Known and predicted interactions of dysregulated genes in mTOR pathway. Non-parametric unpaired Mann–Whitney *t*-test used for comparisons between healthy donors and RA patients. *p < 0.05, ^**^p < 0.01, ^****^p < 0.0001.

Transcriptomics analysis of RNA-Seq on synovial tissue biopsies from healthy donors (*n* = 28) and RA patients (*n* = 151) (GEO database GSE89408) (29) revealed enrichment in several key components in the mTOR pathway (Figures 1A, B) with known and predicted interactions of dysregulated genes depicted in Figure 1C. Specifically, a significant increase in the expression of MTORC1 activators, *LAMTOR2* (*p* < 0.01), *LAMTOR3* (*p* < 0.0001), *LAMTOR5* (*p* < 0.01) was demonstrated between RA synovial tissue compared to healthy donor tissue. *SLC38A9*, an arginine sensor for mTORC1 is also significantly increased in RA compared to healthy donors (*p* < 0.0001) while genes involved in MTORC1 re-localization (*RRAGC* and *STRADB*) were also significantly increased in RA (*p* < 0.0001 and *p* < 0.05, respectively) compared to that of healthy donor tissue. Furthermore, a significant decrease in the expression of *AKT1* and *AKT2*, in addition to *LAMTOR1* (all *p* < 0.0001) (Figures 1A, B), was reported in RA synovial tissue compared to healthy donors. Furthermore, a significant enrichment of several of these genes was demonstrated in individuals at risk (IAR) of developing RA—specifically *LAMTOR2* (*p* < 0.01), *LAMTOR3* (*p* < 0.0001), *LAMTOR5* (*p* < 0.05), *SLC38A9* (*p* < 0.0001), and *RRAGC* (*p* < 0.001). Concomitantly, we also demonstrated significant decreases in the expression of *AKT1* (*p* < 0.0001), *AKT2* (*p* < 0.0001), and *LAMTOR1* (*p* < 0.05) in IAR compared to controls, indicating that mTOR dysregulation may occur within the synovium before clinical manifestations of the disease develop (Supplementary Figure 1). Moreover, following stratification of patients into early versus established disease (early RA being defined as within 12 months of disease diagnosis without prior small or large molecule disease modifying antirheumatic drugs (DMARDs) usage while established RA had received small molecule DMARDs or anti-TNFα treatments), we demonstrated significant dysregulation of several mTOR components in both early and established RA (*LAMTOR3, SLC38A9, RRAGC, AKT1, AKT2, LAMTOR1*—Supplementary Figure 1). Moreover, upon examination of tissue from patients with low disease activity (DAS28 < 3.2) compared to high disease activity (> 5.1), no significant changes in mTOR pathway expression was detected. While this may be indicative that mTOR is regulated throughout disease progression it may not be sensitive to scores of disease activity (data not shown). However, future work should aim to explore the role of mTOR expression compared to degrees of synovitis which may be more reflective of site-specific disease activity than DAS28. Collectively, this reported dysregulation of components of mTOR signaling in RA synovial tissue suggests involvement of the mTOR pathway in RA disease pathogenesis.

### Targeting mTOR pathway in RA synovial fibroblasts alters cellular bioenergetics

**FIGURE 2:**
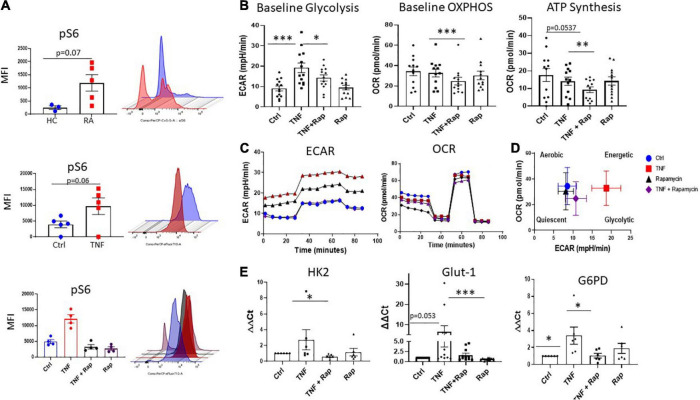
Inhibition of mTOR in RA synovial fibroblasts alters cellular metabolism. **(A)** Representative bar graphs showing MFI expression of the downstream mTOR marker pS6 in healthy donors (*n* = 3) and RA patients (*n* = 5) as well as pS6 expression in RASF (*n* = 5) treated with TNFα (1 ng/ml) for 24 h compared to basal control and RASF (*n* = 4) treated with TNFα (1 ng/ml) and Rapamycin (100 nM) alone or in combination for 24 h. **(B)** Representative bar graphs demonstrating baseline glycolysis, baseline OXPHOS (oxidative phosphorylation), and ATP synthesis, in RASF treated with TNFα (1 ng/ml) and Rapamycin (100 nM) alone or in combination for 24 h (*n* = 12–13). **(C)** Average seahorse profiles demonstrating extracellular acidification rate (ECAR) (glycolysis) and oxygen consumption rate (OCR) (oxidative phosphorylation), before and after injections of oligomycin, FCCP, and antimycin A and rotenone in RASF. **(D)** Metabolic phenotype profiles in RASF representing changes in metabolic phenotype in response to TNFα (1 ng/ml) and Rapamycin (100 nM) alone or in combination for 24 h. **(E)** Bar graphs demonstrating mRNA gene expression of hexokinase 2 (HK2), glucose-6-phosphate dehydrogenase (G6PD), and glucose transporter 1 (GLUT-1), in RASF (*n* = 6–11). Fold increase compared to endogenous controls (RPLPO and HPRT1). Values expressed as mean ± SEM, Mann–Whitney *t*-test used for comparisons between healthy donors and RA patients. Wilcoxon signed rank test used for RASF, *p < 0.05, ^**^p < 0.01, ^***^p < 0.001.

Our data demonstrates dysregulation of the mTOR pathway in RA synovial tissue, therefore, we next determined the cellular source of this dysregulation. RA synovial fibroblasts (RASF) analyzed *ex vivo* from synovial tissue biopsies displayed significantly increased expression of phospho-ribosomal protein S6 (pS6), the major downstream target and effector of the mTOR pathway, in respect to HC (Figure 2A; *p* = 0.07). Furthermore, we can recapitulate this phenotype in *in vitro* cultured RASF following stimulation with TNFα which significantly increases the phosphorylation of S6 (Figure 2A; *p* = 0.06). While there was no significant increase in the protein levels of mTOR (Supplementary Figure 2), increased S6 phosphorylation in RASF demonstrates an increase in mTOR signaling. Furthermore, we evaluated the specificity of Rapamycin to abrogate mTOR signaling in TNFα stimulated RASF and demonstrate a significant reduction in TNFα induced pS6 in the presence of Rapamycin (Figure 2A). As a master regulator of cellular metabolism, we next evaluated the effect of mTOR inhibition on RASF cellular metabolism. Using Seahorse XF96 Technology we simultaneously measured the two major cellular energy pathways, oxidative phosphorylation and glycolysis. We demonstrated a significant reduction in TNFα induced glycolysis (Figure 2B; *p* < 0.05) and oxidative phosphorylation (Figure 2B; *p* < 0.001) in the presence of Rapamycin in RASF. To further examine mitochondrial function in TNFα stimulated RASF in response to mTOR inhibition, a Mito Stress test was performed, thus allowing for multiple parameters of mitochondrial function to be assessed simultaneously in real time. Figure 2C demonstrates the average bioenergetic traces for OCR and ECAR in RASF before and after injections of the mitochondrial inhibitors oligomycin, trifluorocarbonylcyanide phenylhydrazone (FCCP), and antimycin A and rotenone following TNFα stimulation in the presence/absence of Rapamycin. Oligomycin acts as an inhibitor to ATP synthase and is used to evaluate the maximal glycolytic capacity. FCCP which acts a mitochondrial uncoupler was injected to evaluate the maximal respiratory capacity. We report a significant decrease in ATP synthesis in response to TNFα stimulation in the presence of Rapamycin (Figure 2B; *p* < 0.01). However, we report no significant changes in TNFα induced maximal and spare respiratory capacity, proton leak, or coupling efficiency in response to Rapamycin (Supplementary Figure 3). Taken together, these reported changes in glycolysis and oxidative phosphorylation suggest a distinct metabolic shift toward oxidative phosphorylation, away from glycolysis following mTOR inhibition (Figure 2D). In support of this, we also demonstrate significant changes in the expression of several glycolytic genes in response to Rapamycin. Specifically, we demonstrate a significant decrease in the expression of TNFα induced HK2 and G6PD (Figure 2E; *p* < 0.05) in the presence of Rapamycin, while also demonstrating a significant reduction in the expression of TNFα induced GLUT-1—the main glucose transporter in RASF, in the presence of Rapamycin. Collectively these data suggest that mTOR inhibition decreases cellular glycolysis and oxidative phosphorylation in RASF.

### MTOR inhibition in RA synovial fibroblasts decreases cellular migration

**FIGURE 3:**
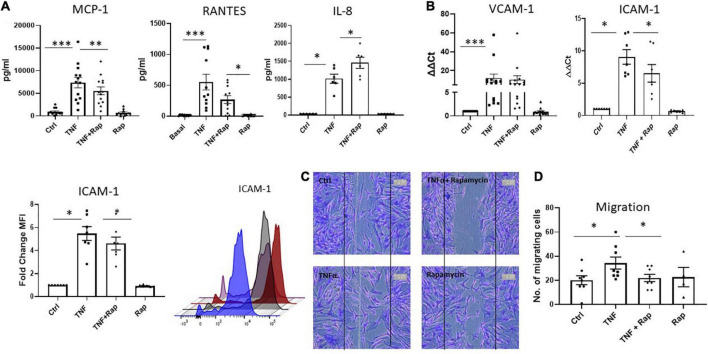
mTOR inhibition in RA synovial fibroblasts decreases cellular migration. Bar graphs showing the measured secretion of **(A)** MCP-1, RANTES, and IL-8 from RASF (*n* = 6–13) and mRNA expression of **(B)** VCAM-1 and ICAM-1 in RASF (*n* = 7–14) treated with TNFα (1 ng/ml) and Rapamycin (100 nM) alone or in combination for 24 h. Gene expression analysis quantified using Real-time PCR. Fold increase compared to endogenous controls (RPLPO and HPRT1). Fold change MFI expression of ICAM-1 on RASF (*n* = 7). **(C)** Representative photomicrographs and **(D)** accompanying bar graph demonstrating cells repopulating the wound to represent migration. Images were taken of RASF (*n* = 8) after being treated with TNFα (1 ng/ml) and Rapamycin (100 nM) alone or in combination for 24 h. Values expressed as mean ± SEM, Wilcoxon signed rank test used for RASF. *p < 0.05, ^**^p < 0.01, ^***^p < 0.001.

It is now widely appreciated that cellular metabolism is intrinsically linked to cellular function. Given that one of the most potent and pathogenic functions of RASF is to migrate to the articular cartilage and invade bone, we next assessed if altered metabolism *via* mTOR inhibition would have functional effects for RASF migration. Firstly, we examined the expression of the chemokine receptors CXCR3, CXCR4, and CXCR5 and noted that while their expression is induced in response to TNFα, Rapamycin does not significantly alter this induction (Supplementary Figure 4). However, we next demonstrate that Rapamycin significantly decreases TNFα induced MCP-1 and RANTES chemokine production (Figure 3A; *p* < 0.01 and *p* < 0.05, respectively) in RASF while simultaneously significantly inducing the production of IL-8 (*p* < 0.05). We also report a significant decrease in TNFα induced expression and production of ICAM-1 in the presence of Rapamycin (Figure 3B; *p* < 0.05). Furthermore, we demonstrate alterations in RASF cellular migration in the presence of Rapamycin. Figure 3C depicts representative images of RASF migration with quantitation depicted in Figure 3D, demonstrating a significant reduction in TNFα induced migration in the presence of Rapamycin (*p* < 0.05).

### MTOR inhibition in RA synovial fibroblasts decreases cellular Invasion

**FIGURE 4:**
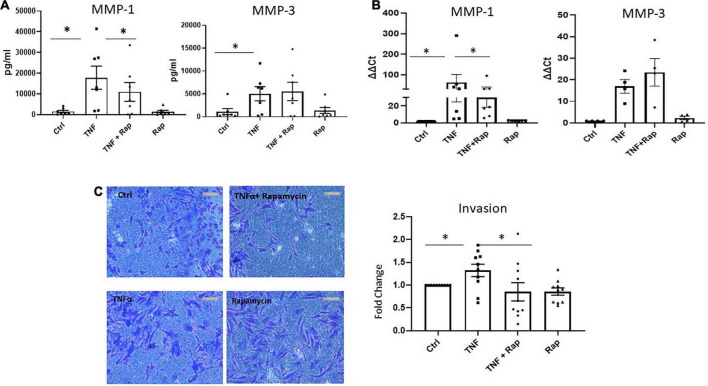
mTOR inhibition in RA synovial fibroblasts decreases cellular invasion. **(A)** Bar graphs showing the measured secretion of MMP-1 and MMP-3 from RASF cultured in the presence of TNFα (1 ng/ml) and Rapamycin (100 nM) alone or in combination for 24 h (*n* = 7). **(B)** Bar graphs showing gene expression of MMP-1 and MMP-3 from RASF (*n* = 4–7). Gene expression analysis quantified using Real-time PCR. Fold increase compared to endogenous controls (RPLPO and HPRT1). **(C)** Representative photomicrographs of RASF invasion along with accompanying bar graph quantifying invasion (*n* = 10). Following 48 h stimulation, invading cells attached to lower membrane were fixed (1% paraformaldehyde) and stained (0.1% crystal violet). Values expressed as mean ± SEM, Friedman Test with Dunn’s multiple comparison and Wilcoxon signed rank test used for RASF. *p < 0.05.

Following migration to the articular cartilage, RASF produce matrix degrading enzymes such as MMPs which facilitates the invasion of RASF into adjacent bone resulting in bone destruction and joint damage. We demonstrate that Rapamycin significantly reduces the expression and production of TNFα induced MMP-1 in RASF (Figures 4A, B; both *p* < 0.05) without altering the expression of MMP-3. Functionally, upon examination of RASF invasion *via* Transwell invasion chambers we also report a significant reduction in TNFα induced invasion in the presence of Rapamycin (Figure 4C; *p* < 0.05). Collectively, these data suggest that mTOR inhibition can reduce the migratory and invasiveness of RASF.

### Crosstalk between dysregulated Hippo-YAP and MTOR pathways mediates RASF invasion

**FIGURE 5:**
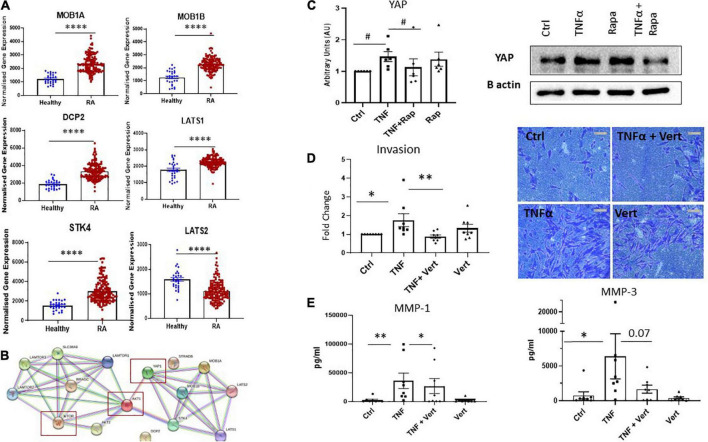
Crosstalk between dysregulated Hippo-YAP and mTOR pathways mediates RASF invasion. **(A)** Bar graphs demonstrating normalized gene expression of MOB1A, MOB1B, DCP2, LATS1, STK4, and LATS2 in RA patients (*n* = 152) compared to healthy donors (*n* = 28). **(B)** Predicted interactions of dysregulated YAP associated genes with dysregulated genes within mTOR pathway using STRING pathway analysis. **(C)** Representative western blot images and bar graph showing total YAP expression in RASF (*n* = 6) treated with TNFα (1 ng/ml) and Rapamycin (100 nM) alone or in combination for 24 h, B-actin was used as a loading control. **(D)** Representative photomicrographs and accompanying bar graph demonstrating invasion and average number of invading cells in RASF (*n* = 8) treated with TNFα (1 ng/ml) and Verteporfin (50 nM) alone or in combination for 48 h. **(E)** Bar graphs showing measured secretion of MMP-1 and MMP-3 from RASF (*n* = 8) treated with TNFα (1 ng/ml) and Verteporfin (50 nM) alone or in combination for 24 h. Values in graphs expressed as mean ± SEM. Non-parametric unpaired Mann–Whitney *t*-test used for comparisons between healthy donors and RA patients. Friedman Test with Dunn’s multiple comparison and Wilcoxon signed rank test were utilized for RASF. *p < 0.05, ^**^p < 0.01, ^****^p < 0.0001, and ^#^*p* < 0.05 ANOVA.

While the mTOR pathway is known as a master regulator of cellular metabolism, its effects on cellular migration and invasion are less well known. Moreover, given our data reports specific effects on these functional phenotypes we hypothesize that mTOR may interact with the Hippo-YAP pathway to mediate this RASF phenotype. Indeed, it is widely appreciated that the YAP pathway is a master regulator of cytoskeleton rearrangement, cell motility and cell invasion. To support this hypothesis, we identified dysregulated expression of several key genes involved in the YAP pathway in RA synovial tissue compared to healthy donor synovial tissue (Figure 5A). Specifically, we report increased expression of *MOB1A, MOB1B, DCP2, LATS1*, and *STK4* (all *p* < 0.0001) in RA synovial tissue with a concomitant significant decrease in *LATS2* (*p* < 0.0001) in RA synovial tissue compared to healthy donor tissue (Figure 5A). Furthermore, deeper analysis of YAP dysregulation across RA disease initiation and progression revealed that several components of the YAP pathway are significantly dysregulated pre-disease in individuals at risk of developing RA—IAR. Specifically, we noted a significant increase in MOB1A (*p* < 0.0001), MOB1B (*p* < 0.005), DCP2 (*p* < 0.0001) with a concomitant decrease in LATS2 (*p* < 0.0001) compared to healthy synovial tissue (Supplementary Figure 5). Moreover several YAP associated genes are also upregulated in undifferentiated arthritis - MOB1A (*p* < 0.001), MOB1B (*p* < 0.0001), DCP-2 (*p* < 0.01), and LATS1 (*p* < 0.0001), early RA - MOB1A (*p* < 0.0001), MOB1B (*p* < 0.0001), DCP-2 (*p* < 0.0001), LATS1 (*p* < 0.0001), and STK4 (*p* < 0.0001), and established RA - MOB1A, (*p* < 0.0001), MOB1B (*p* < 0.0001), DCP-2 (*p* < 0.0001), LATS1 (*p* < 0.0001), and STK4 (*p* < 0.0001) (Supplementary Figure 5). Using STRING pathway analysis, we demonstrate the predicted interactions of these YAP associated dysregulated proteins with those dysregulated within the mTOR pathway and report that both pathways are predicted to interact and converge (Figure 5B). To further support this hypothesis, we investigated the expression of YAP in response to mTOR inhibition with Rapamycin. Specifically, we demonstrate a significant decrease in TNFα induced YAP expression in response to Rapamycin (Figure 5C; *p* < 0.05) suggesting crosstalk between the YAP and mTOR pathways in which mTOR may regulate YAP expression. Finally, we demonstrate that in the presence of a specific YAP inhibitor Verteporfin, there is a significant effect on RASF invasion. Specifically, we demonstrate a significant decrease in TNFα induced RASF invasion in the presence of Verteporfin (Figure 5D; *p* < 0.01) with a concomitant decrease in TNFα induced MMP-1 and MMP-3 expression (Figure 5E; *p* < 0.05 and *p* = 0.07, respectively). Furthermore, we demonstrated that Verteporfin does not significantly affect RASFC viability. Collectively this data suggests that dysregulation in the mTOR pathway in RASF may mediate its affects *via* the Hippo-YAP pathway to induce RASF invasive mechanisms in the joint.

## Discussion

In this study we report a pathogenic role for the mTOR pathway in RA disease progression specifically at the site of inflammation in RA synovial tissue. Specifically, RA synovial tissue has dysregulated expression of several key components of the mTOR pathway. Moreover, we report a specific increase of phosphorylated S6, the major downstream target of mTOR, in RA synovial fibroblasts examined *ex vivo* compared to healthy tissue. We can recapitulate this *in vivo* phenotype with RASF grown in culture stimulated with TNFα which also increase S6 phosphorylation and are responsive to mTOR inhibition *via* Rapamycin. Rapamycin effectively alters RASF cellular bioenergetics by inhibiting glycolysis and the expression of rate limiting glycolytic enzymes. We demonstrate a key role for mTOR signaling in uniquely mediating RASF migratory and invasive mechanisms, which are significantly abrogated in the presence of Rapamycin. Finally, we report a significant upregulation in several genes involved in the Hippo-YAP pathway in RA synovial tissue, which are predicted to converge with the mTOR pathway. We demonstrate crosstalk between the mTOR and YAP pathways in mediating RASF invasive mechanism whereby Rapamycin significantly abrogates YAP expression and YAP inhibition significantly inhibits RASF invasiveness. Collectively, this study highlights a previously unexplored role for mTOR in RASF pathology *via* its interactions with the Hippo-YAP pathway.

We report a significant increase in the expression of the mTOR Ragulator complex components, *LAMTOR2, LAMTOR3*, and *LAMTOR5* in RA synovial tissue. Not only does this complex facilitate the activation of mTORC1, but previous studies have also demonstrated a role for the Ragulator complex in cell migration (31). Moreover, we also demonstrate increased expression of *SLC38A9*, a lysosomal amino acid sensor that induces mTORC1 activation (32) in RA synovial tissue. Previous studies have demonstrated that knockdown of SLC38A9 in RASF, decreased expression of TNFα induced TNFSF13B and CXCL11 and increased expression IL-6 and IL-8 suggesting that mTOR *via* SLC38A9, differentially regulates RASF inflammatory programs (17). We also report a significant decrease in *AKT1* and *AKT2* expression, known upstream regulators of mTOR, in RA synovial tissue. Previous studies have demonstrated a role for the AKT pathway as negative regulators of inflammation. Specifically, inhibition of AKT enhances LPS-induced activation of ERK1/2, p38, JNK and NFκB in human monocytes, thus suggesting that AKT signalling may mediate inhibitory mechanisms of inflammation (33). Collectively, our data suggests that dysregulation of the mTOR pathway within the RA synovium may contribute to disease pathogenesis. To further support this hypothesis, Cejka et al. report that inhibition of mTOR, reduced synovial osteoclast formation and protected against local bone erosions in TNF-transgenic mice with inflammatory arthritis (34). Moreover, Karonitsch et al. also demonstrated increased phosphorylated-mTOR staining in RA synovial tissue compared to osteoarthritis tissue (17).

The inflamed RA synovium is composed of several tissue resident (fibroblasts, endothelial cells, and macrophages) and infiltrating cell subsets (T-cells, B cells, macrophages, Dendritic Cells) which collectively potentiate site specific inflammatory responses, resulting in synovial fibroblast activation, cartilage degradation, and bone erosion. Thus, RASF are non-redundant pathological mediators of joint destruction. We therefore examined the mTOR pathway specifically in RASF, whereby we report increased expression of pS6, a marker of mTOR activation in synovial fibroblasts from RA synovial biopsies. Furthermore, RASF stimulated with TNFα *in vitro* recapitulate this *in vivo* phenotype with increased expression of pS6. This observation is in agreement with previous studies which demonstrate increased activation of pS6 in RASF in response to TNFα stimulation (4). Specifically, we demonstrate that this TNFα induced induction of pS6 is dependent on mTOR as pS6 expression is abrogated in the presence of the mTORC1 selective inhibitor Rapamycin.

It is widely accepted that mTOR plays a critical role in cellular metabolism and as such is often referred to as a metabolic master regulator. While a large body of evidence now exists on the role of mTOR in regulating immune cell metabolism (35), our understanding of the metabolic role mTOR has on stromal cells, in particular fibroblasts is largely unknown. Here we demonstrate for the first time a key role for mTOR signaling in RASF cellular bioenergetics. Specifically, Rapamycin inhibits both glycolysis and oxidative phosphorylation in RASF in addition to the ability of RASF to synthesize ATP. In parallel, we report inhibition in TNFα induced HK2, and G6PD expression in response to Rapamycin while also reporting a significant decrease in GLUT-1 expression. We and others have previously determined that RASF undergo metabolic reprogramming in the joint in response to their increased energy requirements. In response to proinflammatory mediators, such as TNFα, TLR signaling, hypoxia, or oxidative stress, synovial fibroblasts display increased glycolysis coupled with increased expression of glycolytic enzymes and glucose transporters (9, 36). Moreover, previous studies have also determined a role for the JAK/STAT, HIF, and AMPK pathways (9, 37–39) in mediating RASF metabolic reprogramming, whereby activation of these pathways in the synovium results in a more energetic RASF phenotype coupled with increased glycolysis. While several pathways have been identified to date which mediate metabolic reprogramming in RASF, our data is the first to demonstrate a key role for mTOR signaling in RASF bioenergetics.

It is now widely accepted that cellular metabolism is intrinsically linked to cellular function. In addition to mediating metabolic reprogramming in RASF, we also demonstrate a key role for mTOR in RASF function—specifically motility and migration. We report a significant decrease in TNFα induced MCP-1 and RANTES production in response to Rapamycin. This is in agreement with previous studies which report a decrease in LPS induced MCP-1 and RANTES from human monocytes in response to mTOR inhibition (40). Interestingly, we also report a significant increase in the production of TNFα induced IL-8 in response to Rapamycin suggesting that mTOR may negatively regulate IL-8 production. Fibroblast migration is also mediated by the adhesion of fibroblasts to the extracellular matrix (ECM) *via* the expression of adhesion markers such as ICAM-1 and VCAM-1. While we reported no statistically significant change in TNFα induced VCAM-1 expression in response to Rapamycin, we observed a significant decrease in TNFα induced ICAM-1 expression in response to Rapamycin. Similarly, Sun et al. also report decreased adherence of macrophages to HUVECs *via* decreased ICAM-1 in the presence of Rapamycin (41). This suggests that mTOR inhibition may reduce RASF migration in part *via* dysregulated chemokine production and adhesion marker expression. In support of this, we also demonstrate a significant reduction in TNFα induced RASF migration in response to Rapamycin. Indeed, in the cancer setting mTOR plays a critical role in the regulation of tumor cell migration, invasion, and metastasis while previous studies have also highlighted a key role for mTOR in endothelial cell, vascular smooth muscle cell, macrophages and granulocyte migration (14, 42–45).

We also report significant decreases in TNFα induced MMP-1 production and RASF invasion in response to mTOR inhibition. In agreement with our study, Laragione et al. report decreased invasion of both Rat and RASF in response to Rapamycin (16). While this study also reported no significant change in the levels of several MMPs in response to Rapamycin, this was carried out on Rat synovial fibroblasts and therefore is not as reflective as the invasive properties of RASF. Collectively, our data suggests that mTOR specifically regulates synovial fibroblast migratory and invasive mechanisms in RA.

We hypothesized that mTOR may regulate this RASF phenotype *via* crosstalk with the Hippo-YAP pathway. In support of this hypothesis, we demonstrated increased expression of several components of the Hippo-YAP pathway in RA synovial tissue compared to healthy donors while also predicting potential protein interactions *via* mTOR and YAP. Moreover, previous studies have demonstrated cross-talk between the Hippo-YAP and mTOR pathways in regulating organ size during development through their combined control of cell growth and proliferation (26). Furthermore, YAP has been shown to upregulate mTOR activity *via* the upregulation of amino acid transporters (26), while the mTOR pathway itself has been shown to control YAP activity through various mechanisms (25, 46, 47).

Specifically, we demonstrate increased expression of key components of the Hippo-YAP pathway involved in cell proliferation (*MOB1A; MOB1B*) in addition to YAP regulation and activation (*LATS1; LATS2; STK4*). This is in agreement with previous studies which report increased expression of YAP/TAZ target genes in RASF compared to osteoarthritis SF (OASF) (19) while YAP expression is increased in synovial fibroblasts in both AIA mice and human RA (23). In addition to previously aforementioned studies, which demonstrate crosstalk between mTOR and the Hippo-YAP pathway, we also report regulation of YAP expression *via* mTOR signaling in RASF. Collectively, our data in addition to previous studies suggest crosstalk between mTOR and YAP in mediating cell specific effects. Indeed, we report that blockade of YAP *via* verteporfin inhibits TNFα induced RASF invasion, MMP-1, and MMP-3 production. Caire et al. previously reported a significant reduction in TNFα and IL-17a induced invasion in the presence of verteporfin in RASF in addition to a specific reduction in MMP-13 production (19). This suggests that inhibition of YAP and mTOR signaling can reverse the aggressive invasive synovial fibroblast phenotype present in RA.

Collectively our data demonstrates a role for mTOR and YAP crosstalk in RA synovial fibroblast pathogenicity. Given that it has previously been reported that synovial inflammation associated with a fibroblast signature (pauci-immune pathotype) is associated with difficult to treat or refractory RA (5, 6), future studies should explore these pathways in refractory RA versus those that respond to treatment. We hypothesize that due to the pathogenic role mTOR plays in RA fibroblast function, this pathway may be more clinically relevant in those patients with this pauci-immune, refractory disease. Indeed, while our analysis examined mTOR and YAP pathways across the evolution of RA (arthralgia, UA, early, and established RA), an additional limitation of this work was the inability to examine patients in disease remission which would no doubt also provide a clearer picture on the role of mTOR in RA disease pathogenicity and synovial fibroblast activation.

While the metabolic and inflammatory effects of the mTOR pathway have previously been explored in both innate and adaptive immune cells, the contribution this pathway makes to fibroblast pathogenicity has yet to extensively studied. We report a significant role for mTOR in driving RASF metabolism and function with a specific effect on RASF migration and invasion. Furthermore, our data reports a potential role for mTOR:YAP crosstalk in mediating these RASF invasive mechanisms. Targeting the mTOR:YAP axis may prove beneficial in preventing RASF pathogenicity and joint destruction.

## Data availability statement

The datasets presented in this study can be found in online repositories. The names of the repository/repositories and accession number(s) can be found below: https://www.ncbi.nlm.nih.gov/genbank/, GSE89408.

## Ethics statement

Ethical approval to conduct this study was granted by St. Vincent’s Healthcare Group Medical Research and Ethics Committee and all patients gave fully informed written consent prior to inclusion. All experiments were performed in accordance with these guidelines and regulations. The patients/participants provided their written informed consent to participate in this study.

## Author contributions

MC, BB, and UF conceived the experimental approach and designed the experiments. BB, MC, VM, MH, CC, JF, and UF performed the experiments, analyzed the data, and prepared the manuscript. DV recruited the patients, analyzed the data, and prepared the manuscript. CS provided the bioinformatic and data analysis support. MC supervised the project. All authors contributed to the writing of the article and approved the submitted version.
