# Supplementary material for: The mammalian target of rapamycin contributes to synovial fibroblast pathogenicity in rheumatoid arthritis
Source: Front Med (Lausanne). 2023 Feb 3;10:1029021. doi: 10.3389/fmed.2023.1029021 (PMC9936094; doi:10.3389/fmed.2023.1029021)
Supplement: Supplementary file 1 [file Data_Sheet_1.PDF]

PARAMETERS	RHEUMATOID ARTHRITIS (n=35)
**Age** (MEAN +/- SD)	52 (+/- 14)
**Gender :** Female	46%
Male	54%
**Treatment**: No medication	46% (16/35)
DMARD	31% (11/35)
Biologics	17% (6/35)
Other	6% (2/35)
**Seropositve:** RF (+)	14% (5/35)
ACPA (+)	6% (2/35)
ACPA (+) RF (+)	51% (18/35)
**DAS28** (MEAN +/- SD)	4.0 (+/- 1.54)

Supplementary Table 1: Clinical Characteristics of RA patients recruited for RASF analysis
