# Supplementary material for: The mammalian target of rapamycin contributes to synovial fibroblast pathogenicity in rheumatoid arthritis
Source: Front Med (Lausanne). 2023 Feb 3;10:1029021. doi: 10.3389/fmed.2023.1029021 (PMC9936094; doi:10.3389/fmed.2023.1029021)
Supplement: Supplementary file 2 [file Data_Sheet_2.pdf]

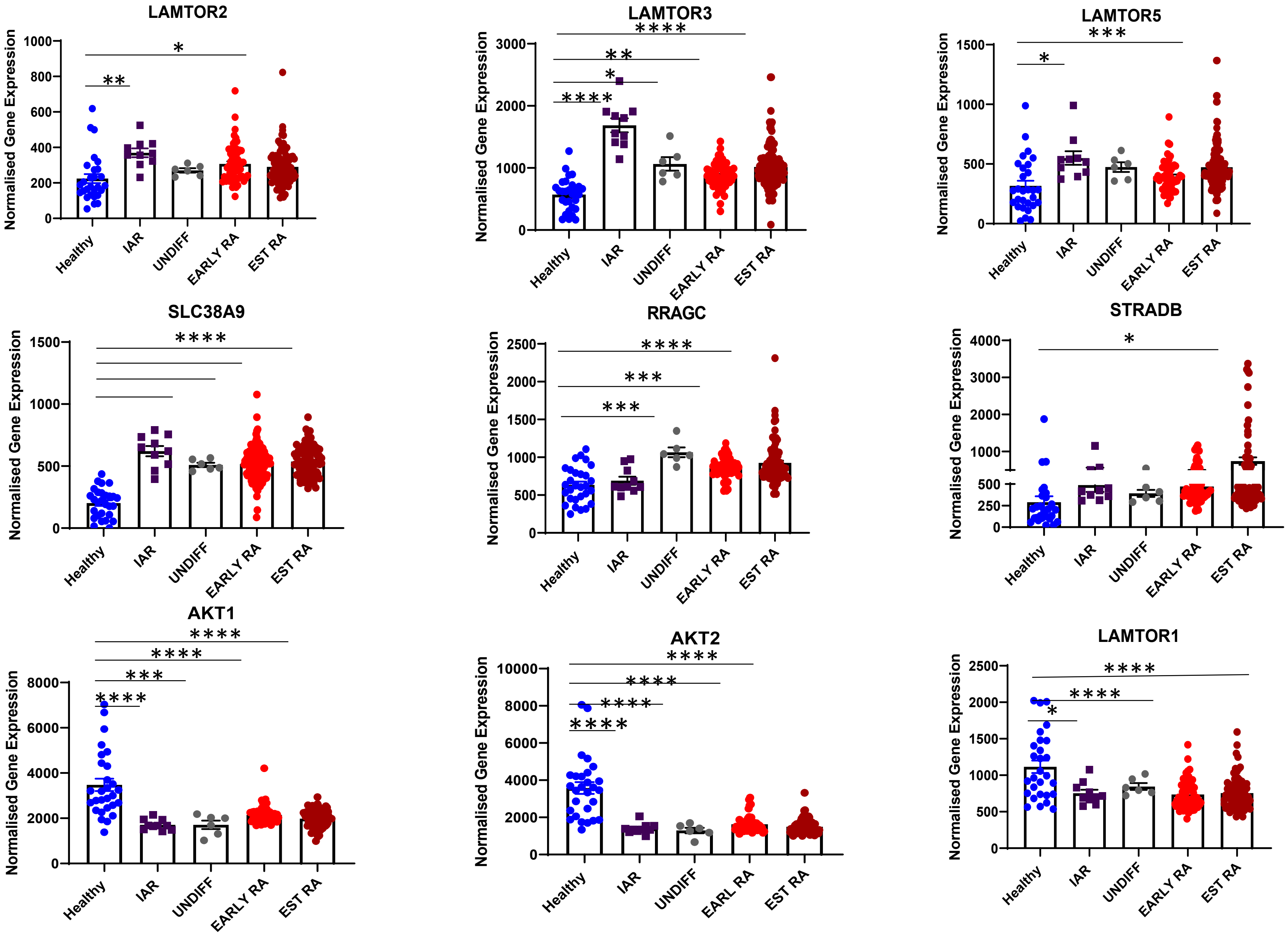

Supplementary Figure 1: Bar graphs demonstrating normalized gene expression of key components in the mTOR pathway (LAMTOR1, LAMTOR2, LAMTOR3, LAMTOR5, SLC38A9, RRAGC, STRADB AKT1, and AKT2) via transcriptomic analysis of RNA-Seq (GEO database GSE89408) on synovial tissue biopsies from healthy donors (n=28), individuals at risk (IAR; n=10), undifferentiatied arthritis (n=6), early RA (n=57) and established RA (n=95)

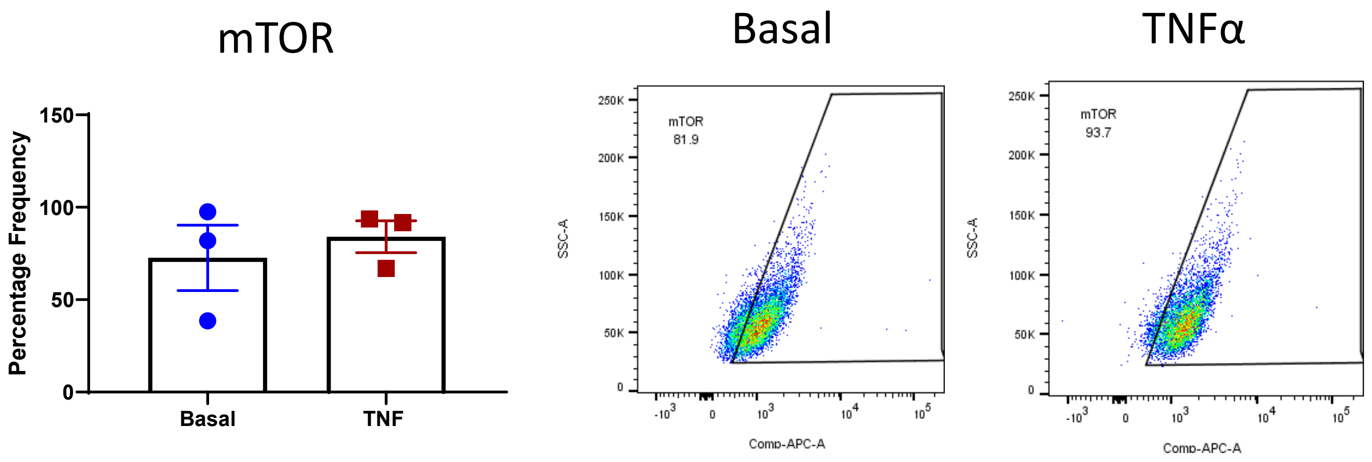

Supplementary Figure 2: mTOR expression in TNFα stimulated RASF. Bar graphs depicting percentage frequency of mTOR positive RASF in the presence or absence of TNFα for 24 hr (n=3). (B) Representative dotplot depicting mTOR positive RASF in the presence or absence of TNFα.

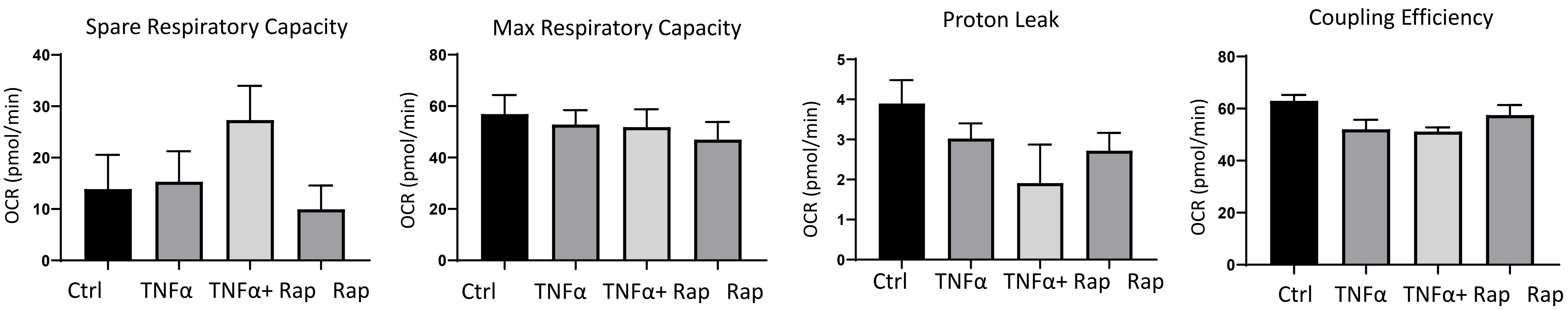

Supplementary Figure 3: Representative bar graphs demonstrating Spare Respiratory Capacity, Max Respiratory Capacity, Proton Leak, and Coupling Efficiency in RASF treated with TNFα (1 ng/ml) and Rapamycin (100 nM) alone or in combination for 24 hr (n=12)

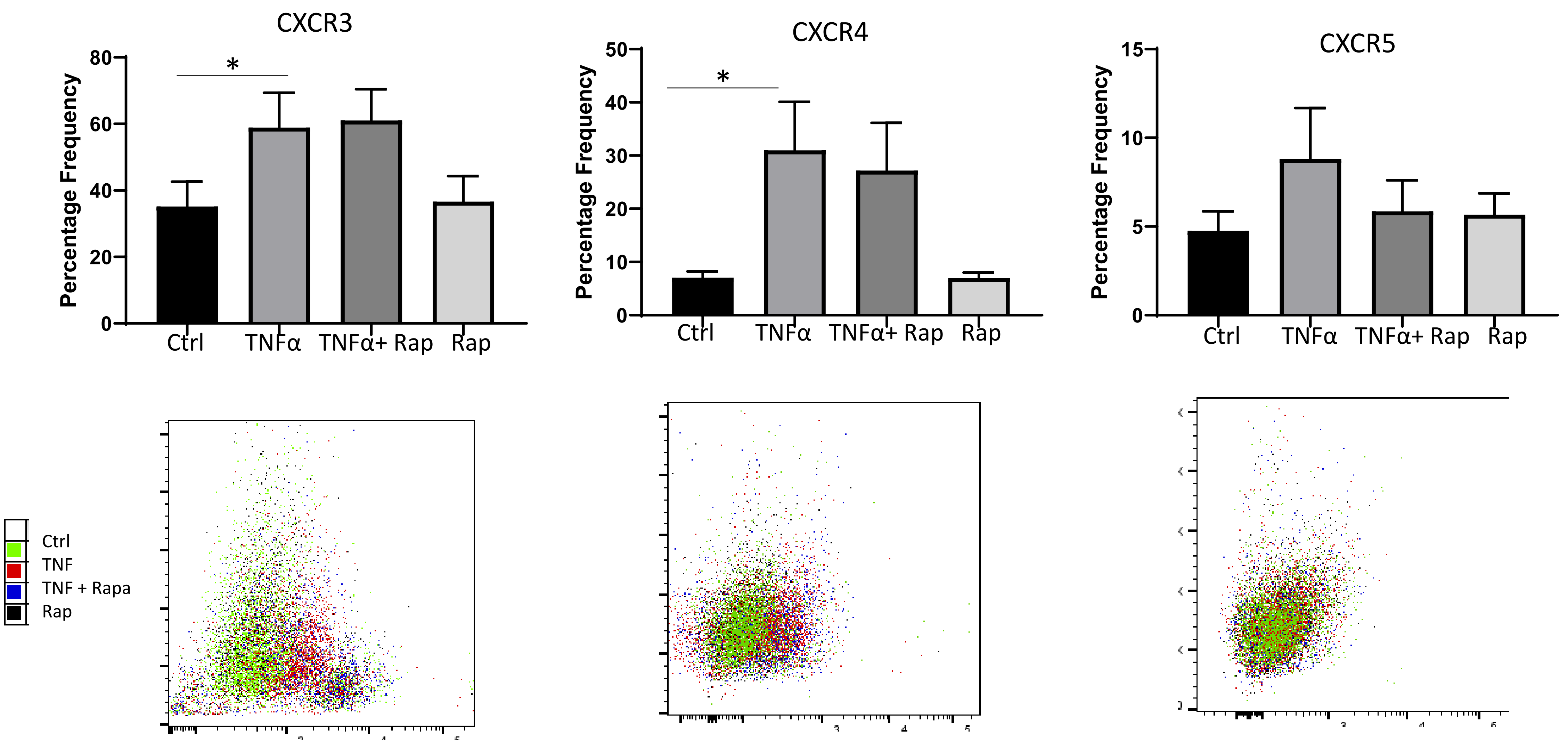

Supplementary Figure 4: (A) Bar graphs demonstrating percentage frequency of CXCR3, CXCR4, CXCR5 positive cells in RASF treated with TNFα (1 ng/ml) and Rapamycin (100 nM) alone or in combination for 24 hr (n=7). (B) Representative dotplots overlaying Ctrl (Green), TNFα (Red), TNFα in the presence of Rapamycin (Blue) and Rapamycin alone (Black)

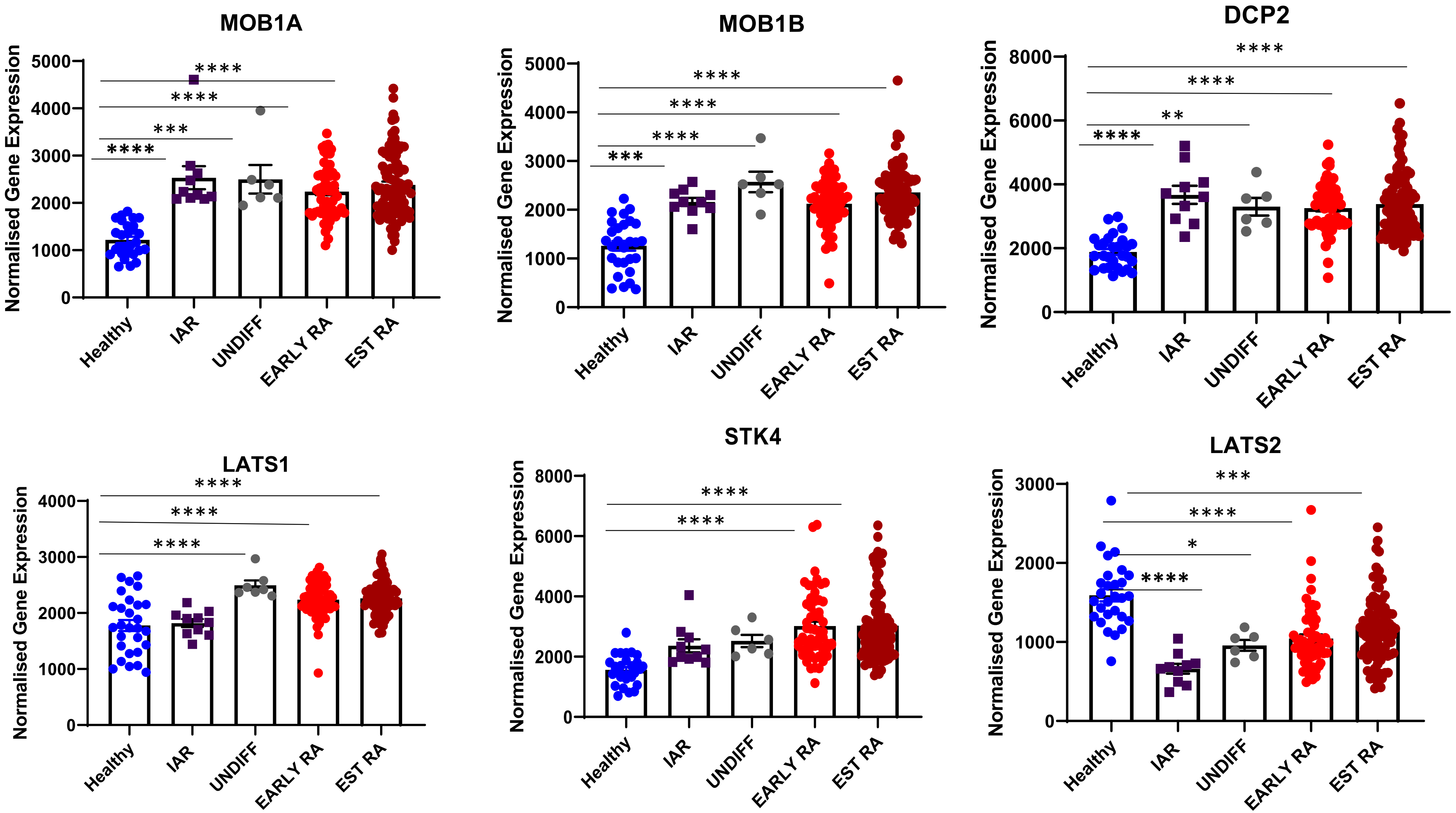

Supplementary Figure 5: Bar graphs demonstrating normalized gene expression of key components in the YAP pathway (MOB1A, MOB1B, DCP2, LATS1 LATS2 STK4) via transcriptomic analysis of RNA-Seq (GEO database GSE89408) on synovial tissue biopsies from healthy donors (n=28), individuals at risk (IAR; n=10), undifferentiated arthritis (n=6), early RA (n=57). Established RA (n=95) and OA (n=22)

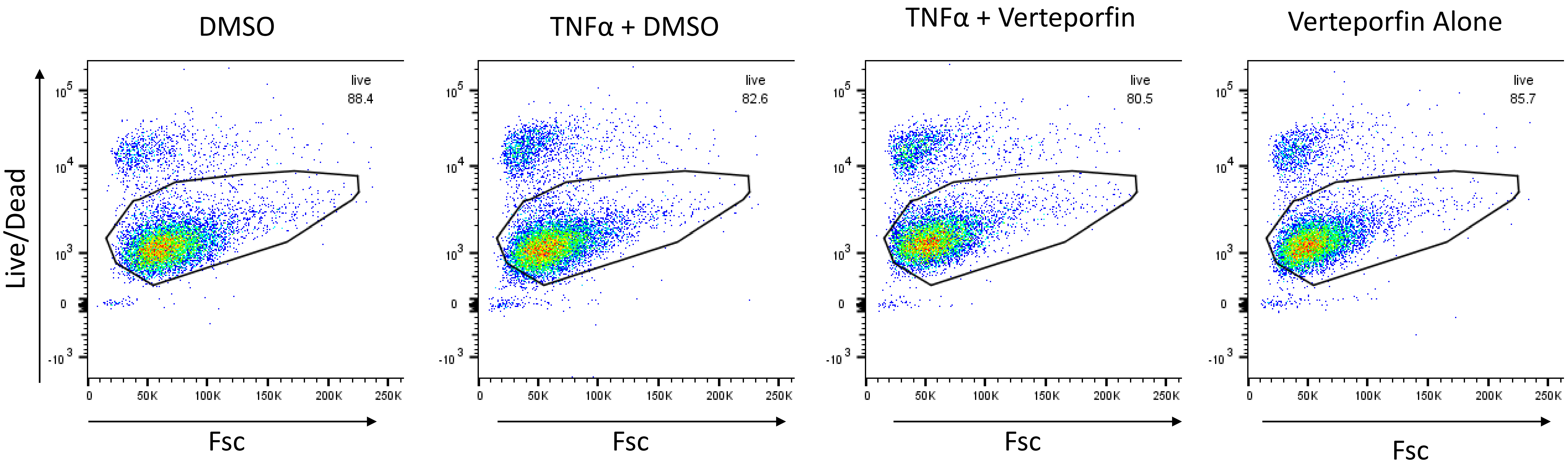

Supplementary Figure 6: Flow Cytometry scatterplots depicting percentage viability of RASFC in response to DMSO, TNFα, TNFα + Verteporfin and Verteporfin alone
